# Characteristics of the 2023-2024 *Mycoplasma pneumoniae* epidemic in adults, Southeast France

**DOI:** 10.1016/j.ijregi.2024.100548

**Published:** 2024-12-18

**Authors:** Anna Wolski, Sophie Edouard, Barbara Melo, Philippe Lavrard, Sébastien Cortaredona, Justine Punturo, Aurélia Bordais, Sami Hraiech, Florence Fenollar, Jean-Christophe Lagier, Nadim Cassir

**Affiliations:** 1 IHU-Méditerranée Infection, Marseille, France; 2 Aix-Marseille Université, AP-HM, SSA, RITMES, Marseille, France; 3 Aix-Marseille Univ, IRD, SSA, MINES, Marseille, France; 4 Aix Marseille Univ, SSA, RITMES, Marseille, France; 5 Service des urgences, Hôpital Nord, AP-HM, Marseille, France; 6 Service de Médecine Intensive - Réanimation, AP-HM, Hôpital Nord, Marseille, France; 7 Faculté de médecine, Centre d'Études et de Recherches sur les Services de Santé et qualité de vie EA 3279, Aix-Marseille Université, Marseille, France; 8 Aix Marseille Univ. AP-HM. MEPHI, Marseille, France

**Keywords:** Mycoplasma pneumoniae, Outbreak, Epidemic, qPCR, Co-infection

## Abstract

**Objectives:**

Analysis of current 2023-2024 *Mycoplasma pneumoniae* (Mp) infection characteristics in adults.

**Methods:**

A retrospective case series analysis was performed on *Mp* polymerase chain reaction-positive adult patients admitted to the University Hospital of Marseille from April 2017 to June 2024. Clinical presentations, treatments, and outcomes were assessed. We compared the epidemiological and clinical characteristics of *Mp* infections between 2017 and 2022 with the current epidemic.

**Results:**

Clinical and radiological characteristics and outcomes of patients with *Mp* infection did not differ significantly between the current epidemic (N = 108) and the 5 previous years (N = 94), except that patients in the current epidemic required less supplemental oxygen (odds ratio [95% confidence interval] = 0.48 [0.29-0.78]) and were less likely to present with fever on admission (odds ratio [95% confidence interval] = 0.22 [0.10-0.47]). In both periods, more than half of the patients hospitalized with *Mp* infection required supportive oxygen therapy.

**Conclusions:**

During the current 2023-2024 epidemic, more hospital admissions for *Mp* infection in adults were observed at the University Hospital of Marseille than in the previous 5 years. The clinical characteristics and outcomes of patients with *Mp* infection did not differ significantly. In our cohort, *Mp* infection was often severe, regardless of the study period.

## Highlights

• *Mycoplasma pneumoniae* hypoxemic pneumonia is frequent.
• Increased access to *M. pneumoniae* polymerase chain reaction testing is needed.
• Clinically, we found slight differences between the current epidemic and before.

## Introduction

*Mycoplasma pneumoniae* (*Mp*) is a common cause of respiratory tract infections with community-acquired pneumonia (CAP) being the major disease burden, most commonly in children aged 5 to 15 years old [1]. Droplets containing the microorganism can be spread from person to person by coughing or sneezing. Infections occur sporadically throughout the year in many different climates around the world, with epidemic waves occurring every few years [1]. Previous data suggest an interval of 1-3 years between *Mp* epidemics in Europe [2]. The reported incidence of sporadic *Mp* in adults ranges from 4-8% of bacterial CAP rising to 20% and 70% during epidemics [3]. Several factors, including waning herd immunity or the introduction of new subtypes into the population, account for the periodic occurrence of epidemics. The previous epidemic occurred in late 2019-early 2020 simultaneously in several countries, mainly in Europe and Asia [4].

Global prospective surveillance data show the re-emergence of *Mp* in Europe and Asia more than 3 years after the introduction of SARS-CoV-2 pandemic restrictions [2,[4], [5], [6], [7]]. The first signs of the recent epidemic were detected in June 2023 and peaked in December 2023 [2]. This increased incidence of *Mp* was observed mainly in school-aged children and young adults [5,6,8,9]. Edouard *et al.* [10] observed a shift in the population affected by the epidemic, with adults becoming more affected from January 2024 (42% of infected patients vs 21% since the beginning of the outbreak), possibly due to massive transmission of the bacterium from infected children. To date, the burden of *Mp* infection in adults is poorly understood. In addition, it remains unclear whether or not the current epidemic is associated with a higher proportion of severe disease.

The aim of this study is to describe the clinical characteristics of *Mp* infections in adults during the current epidemic and to compare them with those of the previous 5 years.

## Materials and methods

Following the STROBE guidelines and checklist, we retrospectively extracted the list of all respiratory specimens tested at the University Hospital of Marseille with one of the following specific quantitative polymerase chain reaction (qPCR) assays for *Mp*: (i) qPCR performed by point-of-care laboratories with a syndromic panel using the Biofire FilmArray Respiratory panel 2 plus assay (Biomérieux, Marcy-l'Etoile, France); (ii) qPCR performed routinely at the core laboratory with a syndromic approach using the FTD Respiratory pathogens 21 assay (Fast Track Diagnosis. Luxembourg) or with an in-house *Mp*-specific qPCR [11]. We did not include those diagnosed by serology because of the lack of immunoglobulin (Ig)M specificity and the long persistence of IgG [10].

The inclusion period started in April 2017 and ended on 31 May 2024. Two periods were defined within this time range: period 1 from 1 April 2017 to 31 March 2023, covering two previous *Mp* outbreaks as well as the beginning of the COVID pandemic, and period 2 from 1 April 2023 to 31 May 2024, covering the current *Mp* epidemic. Only patients over 15 years of age were included. Clinical, biological, and radiological data for each patient were prospectively extracted from our computerized medical record system (DPI Reflex®, aXigate), and pseudonymized.

Study variables included general demographic and medical background data such as age, sex, body mass index, immunosuppression status, and the components of the Charlson comorbidity index. Clinical data at enrollment included vital signs, oxygen flow at enrollment and peak oxygen flow, pulmonary symptoms (cough, sputum), and the presence of extrapulmonary symptoms. Patients were considered to have severe *Mp* if they required oxygen support. Oxygen saturation rate <95% (or 92% in patients with chronic respiratory failure) using pulse oximetry on room air. In addition, we recorded radiological findings, blood test results (erythrocyte, platelet, and leukocyte count, lymphocyte/neutrophil ratio, C-reactive protein level, transaminase, bilirubin, fibrinogen, lactate dehydrogenase, creatinine and urea levels, D-dimer). Finally, outcome data were recorded, including hospitalization, intensive care unit admission, length of hospital stay, and death.

Continuous variables are presented as: mean (standard deviation). Qualitative variables are presented as percentages (n/N). For the univariate analysis, Fisher's exact test was applied to categorical variables, and the Wilcoxon-Mann-Whitney test was used for continuous variables to compare patient groups. To identify the most significant factors associated with the period (1/2), we employed a logistic Bayesian model averaging [12]. Model selection was based on the highest posterior probability and the inclusion of variables in the final model was determined by their posterior inclusion probabilities. Variables with posterior inclusion probabilities greater than 0.5 were considered significant predictors. Initial variable selection was based on univariate analyses, including those with *P* <0.10 as candidates for Bayesian model averaging. Variables with more than 20% missing data or low frequencies were excluded to avoid biases and instability. For the remaining candidate variables, missing values were imputed using multiple imputation techniques via the mice R package (REF). We assessed the missing at-random assumption through both visual analysis and logistic regression models, examining whether the probability of missingness was associated with observed data. The missing at-random assumption was supported by these analyses. Additionally, we performed sensitivity analyses to evaluate the robustness of our findings under different imputation scenarios, confirming the stability of our results. All statistical analyses were performed using R (R Core Team [2023] Foundation for Statistical Computing. Vienna. Austria. URL https://www.R-project.org/).

## Results

**Figure 1:**
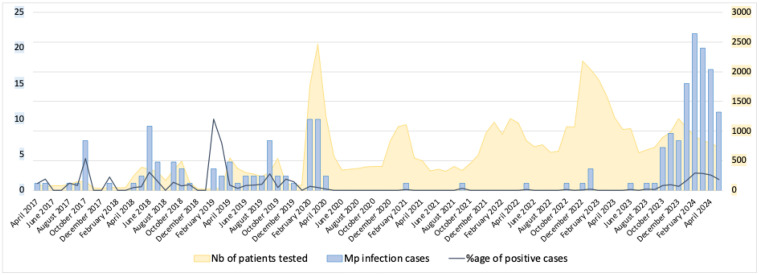
Incidence of *Mp* infection over time from April 2017 to June 2024. *Mp: Mycoplasma pneumoniae*. The number of samples tested over time and the percentage of positive cases are also reported.

**Figure 2:**
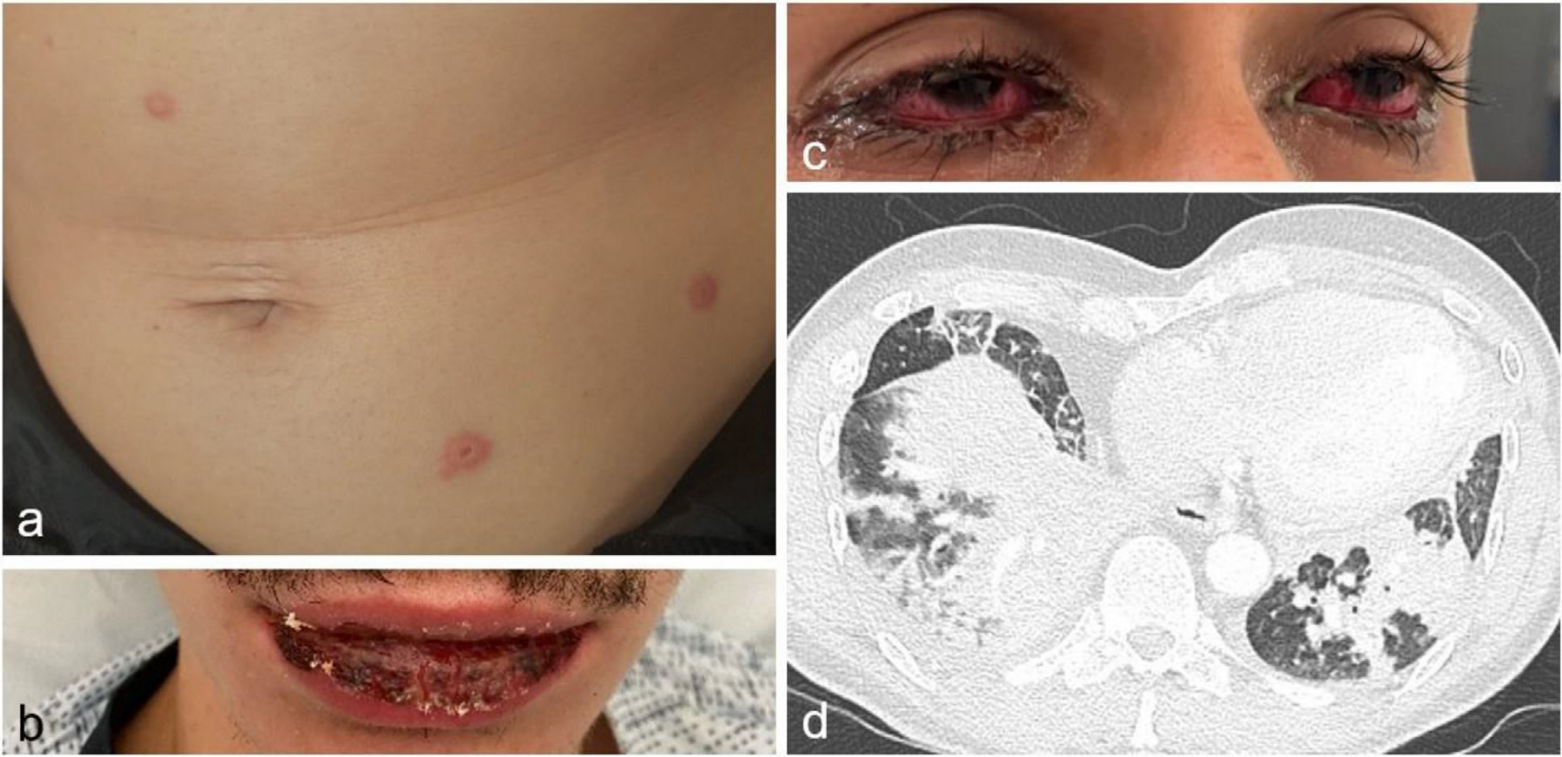
Extrapulmonary and pulmonary presentations of *Mycoplasma pneumoniae* infection. (a) Target-like lesions including a central bulla on the abdomen consistent with erythema multiforme in a 36-year-old female; (b) Mucositis in a 27-year-old male patient; (c) Bilateral conjunctivitis; (d) Bilateral *M. pneumoniae* pleuropneumonia with reactive mediastinal lymph nodes, in a 57-year-old male (axial computed tomographic-scan).

Between April 2017 and June 2024, 118,427 respiratory specimens from 101,548 patients were tested for *Mp* by qPCR at the University Hospital of Marseille. The incidence of *Mp* infection over time is shown in Figure 1. Overall, 535 patients tested positive, of which 202 were 15 years or older, and had available data in the computerized medical record system (Figure S1). The characteristics of the 202 patients are shown in Table 1. The most common clinical presentation of diagnosed *Mp* infection was CAP. Among the 202 included patients, 81.5% presented with at least a cough, 44.4% had sputum expectoration and 31.7% were febrile on admission. Upper respiratory tract (acute coryza, pharyngitis) and gastrointestinal symptoms (nausea, abdominal pain) were also observed in 14.8% and 15.8% of cases, respectively. Mucocutaneous lesions have also been observed in 5.1% of cases (10 patients) consistent with *Mp*–induced rash and mucositis [13] (Figure 2). Other extrapulmonary symptoms were most commonly headache, malaise, and diffuse myalgia (21.4%). More than half of the patients (50.5%) required supportive oxygen therapy, including 6.4% on high-flow therapy, 4.0% on mechanical ventilation, and 3.5% on extracorporeal membrane oxygenation. Most of the patients (74.6%) were admitted to the hospital with a median length of stay of 5 days. The in-hospital mortality rate was 4.45% (9/202) (period 1, N = 7 and period 2, N = 1).

**Table 1:** Characteristics of patients with *Mycoplasma pneumoniae* infection. Comparison of periods 1 (April 2017-March 2023) and 2 (April 2023-May 2024).

	All	Period 1	Period 2		
	N = 202^¤^	N = 94 (46.5%)	N=108 (53.5%)	Missing data^a^	*P*-value^b^
**Baseline characteristics**					
Age (years)	43.4 (22.2)	44.9 (22.3)	42.2 (22.0)	-	0.416
Sex (female)	37.1% (75/202)	36.2% (34/94)	38.0% (41/108)	-	0.884
Body mass index (kg/m²)	25.9 (7.3)	26.3 (7.4)	25.6 (7.3)	74 (37 ; 37)	0.989
Tobacco use	44.0% (84/191)	46.7% (42/90)	41.6% (42/101)	11 (4 ; 7)	0.559
Immunodepression	25.6% (51/199)	30.1% (28/93)	21.7% (23/106)	3 (1 ; 2)	0.195
Charlson comorbidity index	1.2 (1.9)	1.4 (2.1)	1.1 (1.7)	-	0.350
**Clinical presentation**					
Fever (>38°C)	31.7% (64/202)	42.6% (40/94)	22.2% (24/108)	-	0.002
Confusion (Glasgow Coma scale <15)	5.9% (12/202)	4.2% (4/94)	7.4% (8/108)	-	0.388
Oxygen therapy	50.5% (102/202)	43.6% (41/94)	56.5% (61/108)	-	0.090
Heart rate	98.7 (20.3)	94.8 (18.8)	101.9 (21.0)	19 (13 ; 6)	0.012
Cough	81.5% (163/200)	83.7% (77/92)	79.6% (86/108)	2 (2 ; 0)	0.584
Sputum expectorations	44.4% (88/198)	37.4% (34/91)	50.5% (54/107)	4 (3 ; 1)	0.085
Extrapulmonary symptoms	43.1% (87/202)	35.1% (33/94)	50.0% (54/108)	5 (2 ; 3)	0.046
Mucocutaneous	5.1% (10/197)	4.3% (4/92)	5.7% (6/105)	5 (2 ; 3)	0.753
Upper respiratory tract	14.8% (29/196)	13.2% (12/91)	16.2% (17/105)	6 (3 ; 3)	0.687
Digestive	16.8% (33/196)	14.3% (13/91)	19.0% (20/105)	6 (3 ; 3)	0.446
Other	21.4% (42/196)	17.6% (16/91)	24.8% (26/105)	6 (3 ; 3)	0.295
**Outcome**					
Hospitalization	74.6% (151/202)	78.7% (74/94)	71.3% (77/108)	-	0.258
Intensive care unit admission	18.8% (38/202)	22.3% (21/94)	15.7% (17/108)	-	0.280
Length of hospital stay (days)	8.8 (14.5)	10.1 (19.1)	7.6 (8.5)		0.560
Death	4.5% (9/202)	7.4% (7/94)	1.9% (2/108)	-	0.085
**Radiology**					
**Computed tomography-scan features**					
Ground glass opacity	80.6% (83/103)	77.5% (31/40)	82.5% (52/63)	99 (54 ; 45)	0.612
Tree-in-bud	64.2% (61/95)	44.1% (15/34)	75.4% (46/61)	107 (60 ; 47)	0.004
Consolidation	64.6% (73/113)	59.1% (26/44)	68.1% (47/69)	89 (50 ; 39)	0.42
Pleural effusion	15.7% (20/127)	13.2% (7/53)	17.6% (13/74)	75 (41 ; 34)	0.624
Pulmonary embolism	10.3% (7/68)	21.7% (5/23)	4.4% (2/45)	134 (71 ; 63)	0.039
**Microbiology**					
Immunoglobulin M *Mycoplasma pneumoniae* index	13.8 (10.8) 0.6-3.3-9.3-27.0-27.0	10.8 (10.0)	17.6 (10.6)	122 (50 ; 72)	0.009
Co-infections	25.7% (52/202)	26.6% (25/94)	25.0% (27/108)	-	0.872
**Biology**					
Leucocytes (G/l)	10.0 (4.3) 0.9-7.0-9.5-13.0-22.6	10.0 (4.0)	10.0 (4.6)	10 (4 ; 6)	0.941
Lymphocytes (G/l)	1.5 (0.9) 0.0-0.9-1.3-1.9-6.1	1.5 (0.9)	1.5 (1.0)	43 (27 ; 16)	0.827
C-reactive protein (mg/l)	94.4 (81.1)	80.9 (80.0)	105.2 (80.8)	18 (12 ; 6)	0.006

For continuous variables: Mean (SD); For qualitative variables: % (n/N).

a Missing data: Total (N missing for period 1. N missing for period 2).

b Fisher's exact test for frequencies and Wilcoxon-Mann-Whitney test for continuous variables.

Clinical characteristics and outcomes of patients with *Mp* infection did not differ between the periods before (N = 94) and after (N = 108) the COVID-19 pandemic, except that patients in the 2023-2024 outbreak required less supplemental oxygen (odds ratio [OR] [95% confidence interval: CI] = 0.48 [0.29-0.78]), were less likely to present with fever on admission (OR [95% CI] = 0.22 [0.10-0.47]), and their heart rate was significantly higher (OR [95% CI] = 1.93 [1.31-2.83]) (Figure S2). Although, during period 1, the proportion of patients with *Mp* infection having undergone a chest computed tomographic scan was significantly higher (OR [95% CI] = 2.84 [1.35-5.97]), the radiological characteristics were broadly similar between the two periods (Table 1). Co-infections were diagnosed in 25.7% of patients, the most frequent pathogen being rhinovirus, accounting for a quarter of all the co-pathogen detections (Table S1). The rate of co-infection did not differ significantly between both periods.

## Discussion

In this study, we described the epidemiological and clinical characteristics of *Mp* infections in adults during the current 2023-2024 epidemic (period 2) and compared them with those of the previous 5 years (period 1), in a French University Hospital. We observed that more cases of *Mp* infection in adults occurred during period 2 (N = 108) than during period 1 (N = 94). The re-emergence of *Mp* could be explained, at least in part, by the waning herd and individual immunity. Knowing that outbreaks of *Mp* infection occur cyclically every 3-5 years, the current epidemic could be the usual periodic recurrence but marked by an exacerbation due to a period of low exposure to respiratory pathogens because of restrictive measures due to the COVID-19 pandemic. Another explanation could be the emergence of a new *Mp* strain. A recent work suggested that while *Mp* genotypes may not determine specific clinical outcomes, they vary over time and geographic location [14].

While the clinical spectrum of *Mp* infections is diverse, including atypical presentations and potential complications, warranting heightened clinical awareness, we did not observe substantial differences between the current epidemic and the previous ones in our center. In most European countries, current empiric first-line treatment regimens for non-severe CAP mainly consist of beta-lactam antibiotics such as amoxicillin or cephalosporins, which are ineffective against *Mp* [15]. It is therefore imperative that clinicians have a high index of suspicion for atypical micro-organisms causing CAP such as *Mp*, particularly those with atypical clinical presentations or insufficient response to empiric antibiotic treatment for CAP. Increased access to *Mp* PCR testing could facilitate targeted treatment and prevent complications.

We also observed a high rate of co-infection (almost 25%), higher than the 18% reported in the Netherlands [6]. A high rate of co-infection with *Mp* and other respiratory pathogens has been previously described in 65% of children and 34% of adults presenting with acute respiratory infection in the United States [16]. Knowing that asymptomatic carriage could be frequent [17], clinical signs could not be specifically attributed to *Mp* in the case of co-infection. This may explain, at least in part, why more than half of patients hospitalized with *Mp* infection in our study required supportive oxygen therapy, which is higher than in other reports [18].

This study has several limitations. First, this was a single-center retrospective study, so our results could not be generalized. Second, the study population included adult patients assessed in hospitals, not outpatients. Insights into *Mp* infection in children were beyond the scope of the analysis. However, community health management remains critical to pandemic mitigation from a public health perspective. Third, an assessment of drug resistance was not conducted. Reported macrolide resistance is low in Europe, including France [19]. However, caution should be maintained as macrolide-resistance-associated genotypes have emerged as the predominant type in many Asian countries. Given the significance of genotype information in epidemiological investigations, ongoing surveillance is essential.

In conclusion, the clinical spectrum of *Mp* infections is diverse, including atypical presentations and potential complications including hypoxemic pneumonia, warranting heightened clinical awareness. However, in our center, epidemiological and clinical characteristics did not vary substantially between the current epidemic and the 5 previous years. The resurgence of *Mp* post-COVID-19 adds a layer of complexity to respiratory infections. Strategies addressing accurate diagnosis, prudent antibiotic use, and surveillance are critical to managing *Mp* infections worldwide.

## Declarations of competing interest

The authors have no competing interests to declare.

## Appendix Supplementary materials

Image, application 1

Image, application 2

Image, application 3

## Footnotes

Supplementary material associated with this article can be found, in the online version, at doi:10.1016/j.ijregi.2024.100548.
