# Supplementary material for: Characteristics of the 2023-2024 Mycoplasma pneumoniae epidemic in adults, Southeast France
Source: IJID Reg. 2024 Dec 18;14:100548. doi: 10.1016/j.ijregi.2024.100548 (PMC11773249; doi:10.1016/j.ijregi.2024.100548)
Supplement: Supplementary file 1 [file mmc1.docx]

**Figure S1: Flow-chart of the study.**

*Mp* = *Mycoplasma pneumoniae*. qPCR = quantitative Polymerase Chain Reaction.

**N=202**

age $\geq$15

tested after 04/2017

with positive *Mp* qPCR

N=101,548 patients

tested for *Mp*

N=535

with positive *Mp* qPCR

Excluded cases

-Age <15 (N=307)

-Tested before 01/04/2017 (N=26)

-No data available (N=28)

Negative *Mp* qPCR

(N = 101013)

Period 1 : 2017-2023

**N=94**

Period 2 : 2023-2024

**N=108**
