# Supplementary material for: Characteristics of the 2023-2024 Mycoplasma pneumoniae epidemic in adults, Southeast France
Source: IJID Reg. 2024 Dec 18;14:100548. doi: 10.1016/j.ijregi.2024.100548 (PMC11773249; doi:10.1016/j.ijregi.2024.100548)

**Figure S2: Factors associated with period 2 (reference period 1)- Logistic Bayesian Model Averaging - n=202**

period 1: from 1 April 2017 to 31 March 2023

period 2: from 1 April 2023 to 31 May 2024

^a^ In the context of Bayesian Model Averaging (BMA), the coefficients (and hence the odds ratios) for each variable are averaged over all possible models, weighted by the posterior probabilities of these models.

^b^ p-values for the coefficients were computed assuming a normal distribution. This approach is somewhat approximate and not fully Bayesian. In BMA, the concept of p-values is not typically used. Instead, BMA focuses on posterior probabilities of model inclusion (see PIP column).

^c^ Posterior Inclusion Probability (PIP) represents the likelihood of each variable being part of the true model based on the data and priors used in BMA. Variables with PIPs near 1 are strongly supported as important predictors, while those with PIPs near 0 are less likely to be relevant. A PIP > 0.5 is generally considered substantial evidence of inclusion.

Quantitative variables (Heart rate, maximum O2 flow, C-reactive protein level, blood pressure) were standardized (mean = 0, SD = 1). Odds ratios reflect a one standard deviation increase.


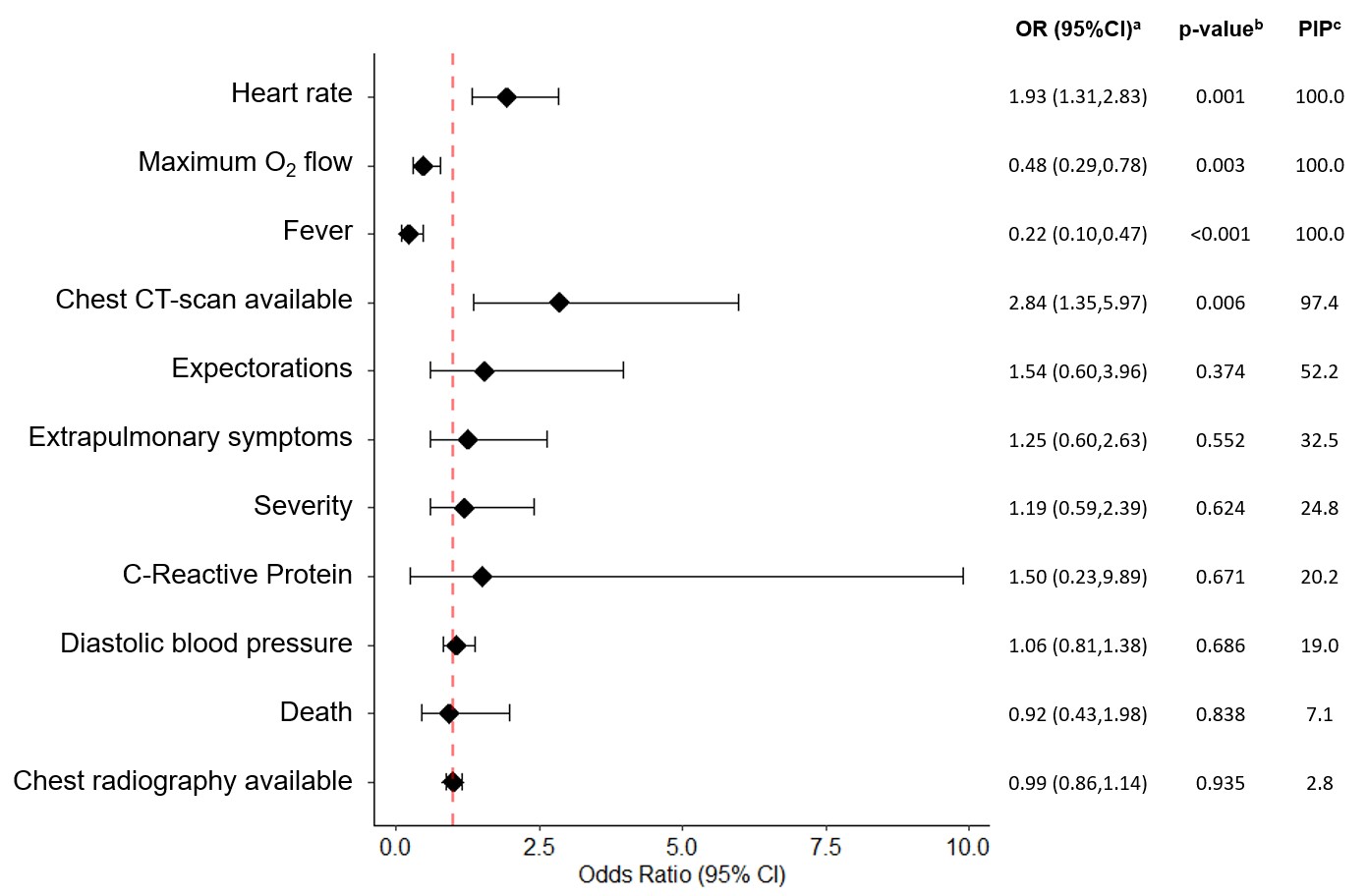

Supplement: Supplementary file 2 [file mmc2.docx]
