# Supplementary material for: Characteristics of the 2023-2024 Mycoplasma pneumoniae epidemic in adults, Southeast France
Source: IJID Reg. 2024 Dec 18;14:100548. doi: 10.1016/j.ijregi.2024.100548 (PMC11773249; doi:10.1016/j.ijregi.2024.100548)
Supplement: Supplementary file 3 [file mmc3.docx]

**Table S1: Microorganisms co-detected on respiratory samples (qPCR and sputum cultures) in patients with *Mycoplasma pneumoniae* infection.**

qPCR = quantitative Polymerase Chain Reaction

| **Pathogen** | **Number of occurrences** |
| --- | --- |
| *Rhinovirus* | 15 |
| *Parainfluenzae3* | 2 |
| *CoronavirusOC43* | 2 |
| *Corona229E* | 2 |
| *Metapneumovirus* | 1 |
| *Adenovirus* | 2 |
| *Bocavirus* | 1 |
| *Influenza B* | 3 |
| *Influenza A H1N1* | 1 |
| *Cytomegalovirus* | 1 |
| *Herpes Simplex Virus* | 1 |
| *Bordetella pertussis* | 2 |
| *Bordetella parapertussis* | 1 |
| *Haemophilius influenzae* | 4 |
| *Streptococcus pneumoniae* | 5 |
| *Pseudomonas aeruginosa* | 4 |
| *Klebsiella* spp. | 2 |
| *Serratia* spp. | 2 |
| *Pneumocystis jirovecii* | 2 |
| *Candida* spp. | 2 |
| *Aspergillus* spp. | 1 |
| *Penicillium chrysogenum* | 1 |
| *Capnocytophaga sputigena* | 1 |
| *Citrobacter koseri* | 1 |
